# The neck burn scar contracture: a concept of effective treatment

**DOI:** 10.1186/s41038-017-0086-8

**Published:** 2017-07-13

**Authors:** Sadanori Akita, Kenji Hayashida, Satoshi Takaki, Yoshihisa Kawakami, Takuto Oyama, Hiroyuki Ohjimi

**Affiliations:** 1 Department of Plastic Surgery, Wound Repair and Regeneration, School of Medicine, Fukuoka University, 7-45-1 Nanakuma, Jonan ku, Fukuoka, 8140180 Japan; 2 Section of Plastic Surgery, School of Medicine, Shimane University, Shimane, Japan; 3 Department of Plastic Surgery, School of Medicine, Fukuoka University, Fukuoka, Japan

**Keywords:** Neck scar contracture, Anatomical locations, Prophylaxis, Severe scar contracture, Thin groin flap, Radiation injury, Adipose-derived stem cell

## Abstract

A neck scar contracture can severely and negatively affect the function of mastication, phonic, or breathing and result in neck pain and issues with esthetics. The best way is of course to avoid such contracture by means of non-surgical treatment such as use of a growth factor. The basic fibroblastic growth factor is clinically well proven in decreasing scar formation and improving healing. There are numerous reconstructive methods for neck contracture, especially when the lesions are relatively limited in part of the neck. However, a very severe and full circumferential scar contracture requires extensive reconstruction. The thin groin flap is one of the answers and well matches with the tissue texture and maintains the flexibility. Even with extensive burns and delayed reconstructions due to resuscitation first, the groin area is well preserved and can be safely harvested by dual vasculature systems of the superficial circumflex iliac artery and superficial epigastric artery, which warrant more reliability compared to the perforator flaps in this area. More demanding and stringent forms of the neck burn scar contracture are the sequelae of radiation. A radiation burn or radiation injury can be progressing and hard to heal. Adipose-derived stem cells can reverse the scar contracture as the surrounding tissue is softened and can accelerate wound healing. In this review, different types of neck burn scar contracture and reconstructive methods are summarized, including innovative use of bFGF and ADSCs in the management of difficult wound healing and scar contracture.

## Background

A neck contracture may result in severe impairment of function and deterioration of esthetics. Children may be progressively worsening and developing further loss of function as they grow and healing is remarkably delayed when wounded. The best way is to avoid a severe burn neck scar contracture by early treatment and mitigations. Once a severe neck burn scar contracture occurs, recovery of the function and esthetics are very difficult.

Generally, the neck burn scar contracture is sub-categorized by anatomical locations and causes of the burn.

Detailed surgical methods to reconstruct the contractures, prophylaxis of the contracture, and novel stem cell therapy are proposed in cases when multiple surgeries fail.

Various approaches using meticulous thin groin flap, the early intervention of a growth factor, and patients’ own stem cells are discussed in the treatment of the neck burn and contracture.

## Review

### Type and reasons for neck scar contracture

#### The anterior neck contracture

Anterior neck contains major functional organ tissues such as the thyroid, carotid, jugular vessels, and the airway. In a series of 11 cases, age 5 to 14 years, post-burn anterior neck contractures were reviewed in an attempt to develop a new approach for reconstruction with local scar-fascial flaps [1].

The severity of the contracture was divided into three grades: (1) mild to moderate, which is of smooth cervicomental angle, restricted neck extension but a normal distance between the chin and the sternal manubrium angle; (2) severe, which is of mouth cervicomental angle and shortened distance between the chin and manubrium; (3) fusion, obliterated chin-manubrium and chin fused with manubrium. The timing of surgery varies from 1 to 5 years after injury. Elimination of the contracture and restoration of the neck contour are the evaluation factors. The benefit in eliminating long and complicated surgical procedures leads to a better outcome of contracture therapy.

Two trapezoid scar-fascial mobilize all components of the anterior neck including scars, fat, platysma, and deep fascia, which enabled elongation of the length from 100 to 200%. It is free from contracture and the cervicomental angle is restored. All the cases gained full functional extensions of the head. The flap is growing, while the reduction of skin grafting of the severe contractures in the submandibular and above the clavicles is minimal. The scar release procedure can exceedingly become more enlarged than planned in advance [2], and the patients with a burn contracture itself necessitate awake fiber-optic intubation, use of intubating laryngeal mask airway, intubation without neuromuscular blocking agents, intubation with neuromuscular blocking agents after testing the ability to ventilate by mask, pre-induction neck scar release under local anesthesia and ketamine, or sedation followed by direct laryngoscopy [3].

#### The lateral neck contracture

In review of the lateral neck contracture, a series of 21 patients of lateral neck contracture, 10 males and 11 females, aging 6 to 43 years old were operated 1 to 3 years after burns, and anatomy and cause of contractures were investigated to determine the type to treat with local flaps. Surgical results were evaluated with functional range of motions and contour. The authors conclude that any contracture is created by the folding, in the areas of which there is an excessive surface and the neighboring surface and anterior neck surface, could be a donor site. Therefore, the local-flaps technique should be considered [4]. In local flaps, either adipose or adipose-scar trapezoid flaps are insisted superior to other local flaps such as V-Y flaps, Z-plasty, triangular flaps in V-Y plasty, or free flaps or pedicle flaps [5–7], when the tissue is excessive in the surface and enables the excessive tissue to be used for scar release. When it is limited, however, the local tissue flaps including the trapeze-flap are not usually considered regardless of the types but the frequency and severity of the lateral neck contractures compared to the anterior neck contracture [4].

#### Severe burns and burn scar contracture of the neck

Severe burn scar contracture after emergency life-saving extensive burn treatment is often observed. There are several types of neck contracture in regard to the depth and the extent of the initial lesion and subsequent secondary effects, but in the most severe cases, the range of motion of the neck was greatly limited and the burn scar contracture extended to the both lateral sides as well as the anterior side, especially, in low- to middle-income countries of resource-limiting situations [8].

#### The neck contracture following radiation injury

Scar contracture after burn injury caused by radiation is especially concerned. Radiodermatitis or radiation injury can be considered a severe form of the burn consequence [9, 10]. Radiodermatitis is categorized in two phases. In the acute phase, it begins with redness or erythema, swelling or edema, and pigmentation. It is often reported with increased cutaneous sensitivity and tightness, which may lead to late (chronic) fibrosis [11]. In greater doses, the patient may develop dry desquamation together with dryness, pruritus, and peeling off of the skin. More increase in dose of ionizing radiation, more frequently moist desquamation is observed.

Months to years after an incident, the chronic phase arises [12]. In chronic phases, changes occur from the edematous skin to hypo- and hyperpigmentation due to damage of dermo-epithelial junction where the melanocyte-melanosome reside. It depends on subjective- and therapy-associated aspects, either prolonging or normalizing with time [13].

Fibrotic tissue changes and excessive capillary formation, telangiectasia, often result from chronic radiation injury, with disposing to ulcers, skin tearing, tissue shrinkage or atrophy and subsequent limitation of motion, pain, and thrombosis obstruction [14]. The tissue is characterized by a marked disruption in healing and increased susceptibility to infection.

Ionizing radiation can penetrate into deeper tissues such as bone, cartilage, muscle, tendon, or ligament. Utilization of ionizing radiation to treat malignant tumors is recently increasing, and other than medical radiation, there are several concerns of “radiation” threat such as nuclear power plant exposure, working exposure to radiation, and weapons.

### The therapeutic methods

#### Promoting wound healing sooner and the use of bFGF

Proper treatment and post-operative care would result in favorable function and esthetics in burn scar treatment. Use of a growth factor such as basic fibroblast growth factor (bFGF) accelerates the burn wound healing in children and adults [15–19] and will minimize scar formation. In view of functional reconstruction, the ideal duration of the bFGF is proposed maximally 3 weeks [3]. The bFGF is effective in minimizing the scars with a dermal component such as a deep dermal component [15, 16, 19] but also brings better scar quality when used in the staged procedure; artificial dermis is preceded for wound beds, which is confirmed both by subjective and objective parameters and measurements [20]. In radiation injury, the tissue may be impaired when increased susceptibility and flap survival may be reversed by the topical administration of bFGF [21]. The topical subcutaneous injection of bFGF improves and maintains the tissue viability after immediate irradiation in the skin and soft tissue [22]. Proper neck garment and fixation device as well as early bFGF treatment may assist better outcome and can be a candidate prevention method for burn neck contracture.

##### Clinical case of the bFGF use

**Fig. 1:**
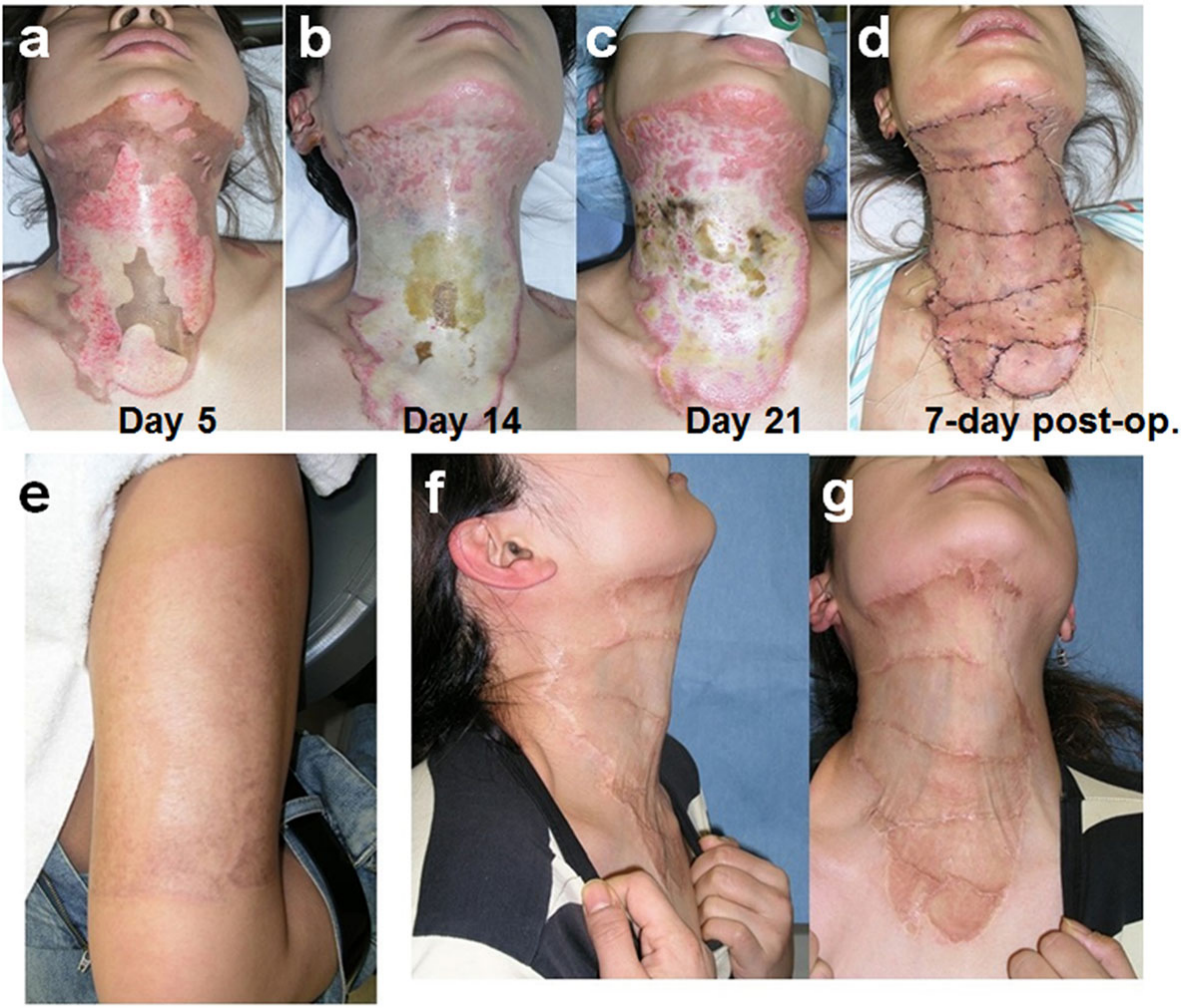
​Clinical case of the bFGF use. A 36-year-old female accidentally flamed her neck while wearing the clothes. The bFGF started from day 2 to day 21 (**a**, **b**, **c**). The removal of eschar and 15/1000-in. split-thickness skin grafting was applied to the neck (**d**). The donor site morbidity is minimal (**e**) and the neck movement is preserved (**f**) although some “picture-frame” slight scars are observed (**g**)

A 36-year-old female was burned by boiled water over her shirts. The burn reached the anterior and lateral surface of the neck. From the day 2, bFGF was started over the deep dermal burn areas and the eschar was removed as much as possible. By day 21, the central area necrosis was demarcated and the debridement and 15/1000-in. split-thickness skin graft from her lateral thigh was grafted (Fig. 1). In 10 months post-operatively, although the “picture-frame” phenomenon slightly remained, the function, rotating, maximal extension and flexion of the neck, and esthetics in less scar contracture and color-match to the neck were acceptable (Fig. 1).

#### Regional strategy for the neck contracture

Severe neck burn contracture deteriorates the range of motion and cosmesis [8]. Many surgical techniques have been attempted and failed to define the therapeutic guideline or algorithm. In review of 24 patients, by the third post-operative month, soft tissue cervico-mandibular (CM) angles significantly decreased, while both osseous CM and dynamic angles defined by full extension and full flexion, are significantly increased. Soft tissue CM angles are considered static markers, and affected by multiple factors and could be a pit fall into masked disfigurement. Either descriptive or quantitative, CM angle is a very important hallmark both for normal and neck contracture [23–27], and once impaired, both function and esthetics are prominent. In order to obtain better outcome, platysma manipulation is stressed to achieve a proximal muscle flap that is returned and stabilized to the border of the lower mandible as this is able to augment the submental region, angulate the soft tissue CM angle, and escape from recurrence of contracture. Additionally, the lower muscle can be turned down to surface the scar region at the distal cervicothoracic junction and facilitate skin graft.

#### Thin and malleable flap reconstruction for severe and extended scar contracture

It is highly important to replace the deficit resulting in the scar contracture by sufficient amount, malleable enough, and color- and texture-matched tissue. When the contracture is limited to moderate to intermediate, neighboring tissue expansion or similar tissue donor-site flap reconstructions are opted [28–31].

**Fig. 2:**
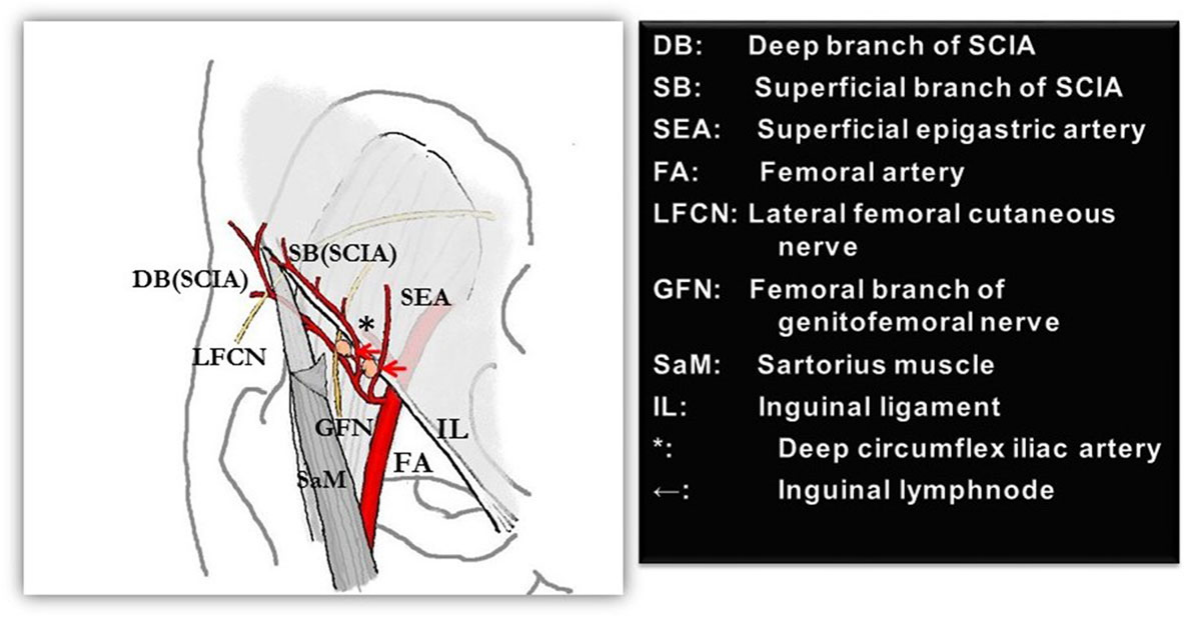
Vascular anatomy of groin region. “Groin flap” can be warranted by dual vasculature of the superficial circumflex iliac artery and superficial epigastric artery

In such severe and peri-full circumferential cervical scar contracture, free (vascularized) flap from the groin was planned. The free groin flap is very soft and less bulky thus adjustable to the defect and easily matched to the surrounding tissue and often preserved as a “donor” site even in severe and extensive burns, it is also considered with very low donor-site morbidity [32] and easily adjustable to the frequent mobile joint such as the head and neck [33]. Also, the superiority of this flap compared to the perforator flap is that two anatomically stable pedicles could be candidate for the pedicle vessels, i.e., the superficial branch of the superficial circumflex iliac artery (SCIA) and superficial epigastric artery (SEA). The groin flap can be tailored to the “thin groin flap” under a microscope before or after cut-off of the nutrient vessels in three fourths of the areas except the vessel, SCIA or SEA, trunks. A 5 × 12 cm to 13 × 30 cm can be harvested and defatting of the flap in order to make it more malleable can be employed in the medial half portion of the flap and in the case of the “short pedicle” method, especially when separating the SCIA and the SEA (Fig. 2). Compared to the perforator flap, which identifies the perforator nutrient flap first [34] and thus is limited in more complex and larger tissue defect coverage, the thin groin flap may be still of use in post-scar contracture release [35].

In severe and extensive burns, which could delay the proper treatment of preserving the neck range of motion and the esthetics of the scar, the groin regions are almost always intact and can be a candidate for functional and esthetic reconstruction.

##### Clinical cases of the thin groin flap

**Fig. 3:**
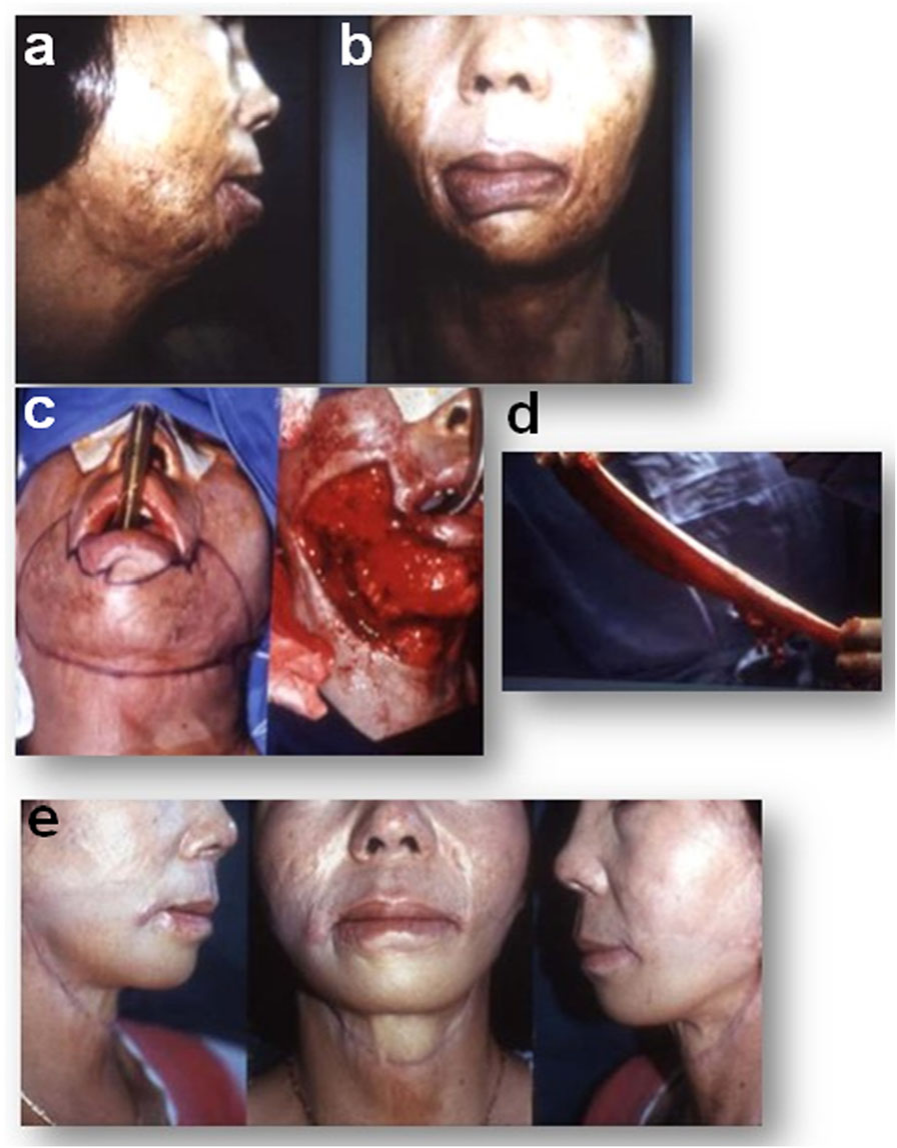
Clinical case of the thin groin flap. A 40-year-old female developed severe lower mandible and neck scar contractures (**a**, **b**). The patient suffered from drooling and lower lip ectropion. Scar release including the lower lip and neck were covered by 10 × 25 cm thin groin flap fitted to the defect (**c**, **d**), and the neck function and appearance improved at 24 months (**e**)

A 40-year-old female suffered from the severe limitation of the neck movement and the mouth movement related to mastication and sometimes drooling because the lower lip was heavy because of ectropion and hard to close. Three years after burn, in order to remove the scar tissue in the inferior lip and mandible, to improve the CM angle, and to replace the anterior neck area, debridement until the facial expression and platysma muscles was performed, and the wound was covered with a 10 × 25 cm wide defatted (thin) groin flap vascularized both SCIA and SEA vasculature. In 24 months post-operation, the mastication improved smoothly and no drooling was observed. Both color and texture matched to the neighboring tissue, and the neck movement and shape of the mandibular area returned close to normal. The facial expression went easier after flap reconstruction (Fig. 3).

**Fig. 4:**
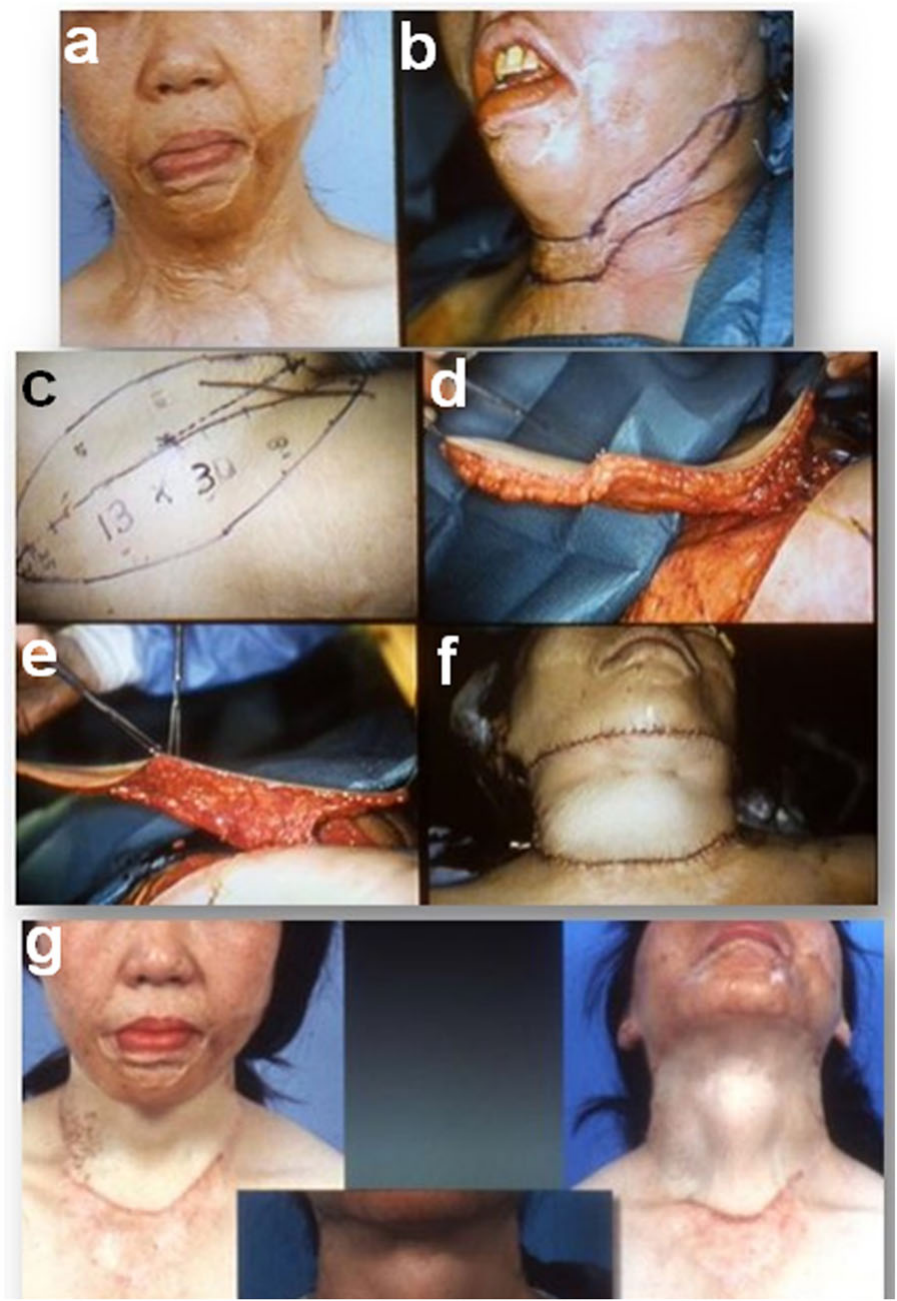
Clinical case of the thin groin flap. A 39-year-old female developed the severe full circumferential neck scar contractures (**a**, **b**). The impaired neck motion as well as lip incompetence led to the severe limitation of her quality of life. A 13 × 30 cm defatted thin groin flap (**c**, **d**, **e**) was set to fill in the cervico-mandibular area and three quarters of the cervix after scar release (**f**). After 24 months post-operation, the neck morphology reflected the thyroidal cartilage and muscles including the sternocleidomastoideus (**g**). The lower lip ectropion is reversed more normally, and lip incompetence was restored

A 39-year-old female with flame burn resulted in extensive burn scar contracture in the lower face and full-length cervical surface in the anterior and lateral parts. A 13 × 30 cm defatted thin groin flap was inset to fill in the cervico-mandibular area and three quarters of the cervix after scar release. After 24 months post-operation, the neck morphology reflected the thyroidal cartilage and muscles including the sternocleidomastoideus. The lower lip ectropion was reversed more normally and lip incompetence was restored (Fig. 4).

#### The chronic sequelae of radiation injury

As the prophylaxis is very hard and mitigation is less idealistic in current clinical modalities [36], novel therapies are innovated and clinically tested. Among them, bone marrow-derived stem cells (BMDSCs) firstly seem to play an integral role in regeneration of tissue. Other than bone marrow-derived, mesenchymal cells are able to lead to the healing process with other progenitor cells such as endothelial progenitor cells, and myelomonocytic cells. In vitro, it is confirmed these cells are drawn to sites of radiation damage due to the chemotactic effects of stromal cell-derived factor and overproduction of CXCR4 [37, 38]. Myelomonocytic cells are available for the predominant BMDC to localize in irradiated tissue and stimulate vascular regeneration and repair through actions of angiogenic and trophic factors. The mesenchymal stem cells including BMDCs and adipose-derived stem cells (ADSCs) are successfully implicated in surgical treatment of difficult and intractable clinical applications, and human ADSCs are beneficial for direct use because of the abundance and diversity of the cell sources [39] and have been shown to be an effective treatment in accelerating the wound healing process [40]. ADSCs are multipotent cells capable of promoting angiogenesis, secreting and interacting with biochemical factors and messengers, and stimulating dermal fibroblast proliferation during the re-epithelialization phase of wound healing [41].

Also, these cells may begin with the inflammatory and modulating cascade and cause ischemia reperfusion injury [42].

Even though there are cell-specific differences at transcriptional and proteomic levels between bone marrow- and adipose-derived stem cell types according to their tissue origin and processes towards adipogenic, osteogenic, and chondrogenic differentiation, in vitro as well as in vivo ADSCs display the same ability as bone marrow-derived stem cells to differentiate towards chondrocytes/osteoblasts, comforting the status of both cell sources as promising regenerative cells [43]. ADSCs are easier to harvest and more abundant and can be distant from the radiations sites [40].

##### Clinical case of the treatment of chronic radiation injury by ADSCs

**Fig. 5:**
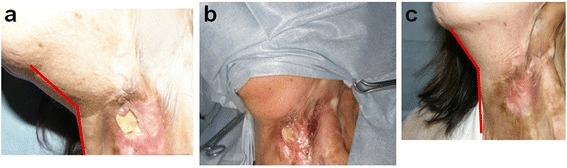
Clinical case of the treatment of chronic radiation injury by ADSCs. A 52-year-old female was suffering from intractable chronic radiation wounds, which limited her neck movement, range of motion of 130° (**a**). The defect was sized 25 × 17 mm and reached deep partially to the exposed left thyroid cartilage (**b**). Non-cultured cells with adipose-derived stem cells were processed and then injected to the debrided wound margin and in the wound base. In 75 days, the wound was healed and the neck forward movement was improved, and at 6 months, the injected subcutaneous lesion has still kept its soft texture and demonstrated the thick and vascularized soft tissue and the range of motion improved to 165° (**c**)

A 52-year-old female was suffering from intractable chronic radiation wounds, which limited her neck movement and the range of motion was 130°. The patient underwent multiple surgeries including skin grafting, staged artificial dermis and skin grafting, local flaps, and free flaps. The wound stayed closed for a short period but always became recurrent within a few months. The subjective limitation of the neck movement never recovered. About 350 ml of adipose tissue was harvested by liposuction procedure. The harvest contained 4.1 × 10^7^ adipose-derived cells including ADSCs. Meticulous sharp debridement was conducted, with carotid artery identified by ultrasonic avoiding damage, which was more anteriorly positioned due to contracture after radiation and previous surgeries. The defect was 25 × 17 mm in size and reached deep partially to the exposed left thyroid cartilage. Cells with ADSCs were injected to the debrided wound margin and in the wound base. In 75 days, the wound was healed and the neck forward movement was improved, and at 6 months, the injected subcutaneous lesion still kept its soft texture and demonstrated the thick and vascularized soft tissue and the range of motion improved to 165° (Fig. 5).

#### Rehabilitation

Positioning and application of pressure to the neck is significantly related to the need for neck reconstruction. Delayed pressure and positioning of the neck after skin grafting can result in an earlier and more frequent need for neck reconstruction [44]. However, a malleable and extensive flap may restore more effectively than skin grafting and may lead to better outcome.

### Discussion

The neck is an integral part of the major organs in breathing, mastication, phonics, and glands of endocrines such as the thyroid and parathyroid and the salivary glands.

Facial expressions can also be determined in the facial muscles in the face and the neck. Once impairment in pediatric period occurred, subsequent effects to motion, growth, and smile are enormous in psychiatric drawback as well as objective impairment [45–47]. In adult extensive burns, the neck might be less attentive as initial treatment is attempted in resuscitation and much easier areas, which may result in the delayed start of treatment and lead to severe contracture although the incidence is lower compared to other joints in extremities [48]. The most important strategy is to avoid the severe neck contracture sequelae by careful treatment in a timely manner. In partial thickness burns, growth factor treatment leads to faster wound closure, less scarring and thus better quality of wound healing [15, 16, 19].

In the severe neck contractures, many reconstructive options are proposed including local [1, 2, 4–7] and free flaps [24] and combination with tissue expansion. However, in very severe cases, the donor sites can be hardly found. The groin area is one of the options as it is large in size, malleable, and adjustable to the recipient scar-released defects and safer with “dual” vascular nutrients by superficial circumflex iliac vessels and superficial epigastric vessels [32, 33].

Radiation injury can progressively and rapidly alter tissue texture and may lead to fibrosis. Starting with radiodermatitis to intractable wounds surrounded by fibrotic less vascularized tissues, radiation injury can result in one of the most difficult burn sequelae [39–41]. Many therapies are attempted to manage radiation injury, and ADSCs are considered a great candidate of the cell therapy as they are multipotent and capable of promoting angiogenesis, secreting and interacting with biochemical factors and messengers, and stimulating dermal fibroblast proliferation during the re-epithelialization phase of wound healing [39].

In neck contracture with ulcer subsequent to chronic radiation injury, the ADSC treatment improved the scar contracture as well as the wound healing [40, 41].

## Conclusions

Although many proposals of reconstructions of the neck burn scar contractures were discussed, the severe cases of full circumferential contractures have not been clarified.

Even in severely extensive burns, the groin areas are primarily preserved and the thinned groin flap, of which nutrient vessels are dual by the superficial circumflex iliac and superficial epigastric vessels, is a good candidate in fully contracted neck scar coverage.

Radiation injury may cause fibrotic tissue changes with intractable wounds in a long term. In certain cases, treatment of the radiation injury-caused neck scars by the patient’s own adipose-derived stem cells can heal the wound in weeks after a single cell therapy with very softened and flexible neck range of motion.
